# Multimodal care for the management of musculoskeletal disorders of the elbow, forearm, wrist and hand: a systematic review by the Ontario Protocol for Traffic Injury Management (OPTIMa) Collaboration

**DOI:** 10.1186/s12998-016-0089-8

**Published:** 2016-03-07

**Authors:** Deborah Sutton, Douglas P. Gross, Pierre Côté, Kristi Randhawa, Hainan Yu, Jessica J. Wong, Paula Stern, Sharanya Varatharajan, Danielle Southerst, Heather M. Shearer, Maja Stupar, Rachel Goldgrub, Gabrielle van der Velde, Margareta Nordin, Linda J. Carroll, Anne Taylor-Vaisey

**Affiliations:** UOIT-CMCC Centre for the Study of Disability Prevention and Rehabilitation, University of Ontario Institute of Technology (UOIT) and Canadian Memorial Chiropractic College (CMCC), 6100 Leslie Street, Toronto, Ontario Canada M2H 3J1; Division of Graduate Education and Research, Canadian Memorial Chiropractic College (CMCC), 6100 Leslie Street, Toronto, Ontario Canada M2H 3J1; Faculty of Rehabilitation Medicine, Department of Physical Therapy, University of Alberta, 8205 114 St, 3-28 Corbett Hall, Edmonton, AB Canada T6G 2G4; Rehabilitation Research Centre, University of Alberta, 8205 114 St, 3-48 Corbett Hall, Edmonton, AB Canada T6G 2G4; Canada Research Chair in Disability Prevention and Rehabilitation, University of Ontario Institute of Technology (UOIT), 2000 Simcoe Street North, Oshawa, Ontario Canada L1H 7L7; Faculty of Health Sciences, University of Ontario Institute of Technology (UOIT), 2000 Simcoe Street North, Oshawa, Ontario Canada L1H 7L7; Division of Undergraduate Education, Canadian Memorial Chiropractic College, 6100 Leslie Street, Toronto, Ontario Canada M2H 3J1; Graduate Education Program, Canadian Memorial Chiropractic College (CMCC), 6100 Leslie Street, Toronto, Ontario Canada M2H 3J1; Rebecca MacDonald Centre for Arthritis and Autoimmune Disease, Mount Sinai Hospital, Joseph and Wolf Lebovic Health Complex, 60 Murray Street, 2nd Floor (Main), Toronto, Ontario Canada M5T 3L9; Graduate Student, Faculty of Health Sciences, University of Ontario Institute of Technology (UOIT), 2000 Simcoe Street North, Oshawa, L1H 7L7 Ontario Canada; Toronto Health Economics and Technology Assessment (THETA) Collaborative, 6th Floor, Room 658, 144 College Street, Toronto, Ontario Canada M5S 3M2; Faculty of Pharmacy, University of Toronto, 144 College Street, Toronto, Ontario Canada M5S 3M2; Institute for Work and Health, 481 University Avenue, Toronto, Ontario Canada M5G 2E9; Departments of Orthopedic Surgery and Environmental Medicine, Occupational and Industrial Orthopedic Center, NYU School of Medicine, New York University, 550 1st Avenue, New York, NY 10016 USA; School of Public Health and Injury Prevention Centre, University of Alberta, 3-300 Edmonton Clinic Health Academy 11405 – 87 Ave, Edmonton, Alberta Canada T6G 1C9

**Keywords:** Wrist injuries, Hand injuries, Carpal tunnel syndrome, Review literature as topic, Tennis elbow, Epicondylitis, Multimodal treatment

## Abstract

**Background:**

Musculoskeletal disorders of the elbow, forearm, wrist and hand are associated with pain, functional impairment and decreased productivity in the general population. Combining several interventions in a multimodal program of care is reflective of current clinical practice; however there is limited evidence to support its effectiveness. The purpose of our review was to investigate the effectiveness of multimodal care for the management of musculoskeletal disorders of the elbow, forearm, wrist and hand on self-rated recovery, functional recovery, or clinical outcomes in adults or children.

**Methods:**

We conducted a systematic review of the literature and best evidence synthesis. We searched MEDLINE, EMBASE, CINAHL, PsycINFO, and the Cochrane Central Register of Controlled Trials from January 1990 to March 2015. Randomized controlled trials, cohort studies, and case–control studies were eligible. Random pairs of independent reviewers screened studies for relevance and critically appraised relevant studies using the Scottish Intercollegiate Guidelines Network criteria. Studies with a low risk of bias were synthesized following best evidence synthesis principles.

**Results:**

We screened 5989 articles, and critically appraised eleven articles. Of those, seven had a low risk of bias; one addressed carpal tunnel syndrome and six addressed lateral epicondylitis. Our search did not identify any low risk of bias studies examining the effectiveness of multimodal care for the management of other musculoskeletal disorders of the elbow, forearm, wrist or hand. The evidence suggests that multimodal care for the management of lateral epicondylitis may include education, exercise (strengthening, stretching, occupational exercise), manual therapy (manipulation) and soft tissue therapy (massage). The evidence does not support the use of multimodal care for the management of carpal tunnel syndrome.

**Conclusions:**

The current evidence on the effectiveness of multimodal care for musculoskeletal disorders of the elbow, forearm, wrist and hand is limited. The available evidence suggests that there may be a role for multimodal care in the management of patients with persistent lateral epicondylitis. Future research is needed to examine the effectiveness of multimodal care and guide clinical practice.

**Systematic review registration number:**

CRD42014009093

**Electronic supplementary material:**

The online version of this article (doi:10.1186/s12998-016-0089-8) contains supplementary material, which is available to authorized users.

## Background

Musculoskeletal disorders, as defined by the Centers for Disease Control and Prevention (CDC), are injuries or disorders of the muscles, nerves, tendons, joints, cartilage and supporting structures of the upper and lower limbs, neck and lower back [1]. These disorders may be caused or exacerbated by exertion or prolonged exposure to physical factors. Musculoskeletal disorders of the elbow, forearm, wrist and hand commonly affect limb function, social activities and the ability to work [2, 3]. In 2014, arm, wrist and hand injuries accounted for 12.0 % of lost time claims in Ontario workers [4]. Musculoskeletal disorders can occur in the supporting ligaments and capsule of the humeroulnar, humeroradial, and proximal radioulnar joints of the elbow, as well as the distal radioulnar, radiocarpal, intercarpal, midcarpal, carpometacarpal and intermetacarpal joints and may involve the triangular fibrocartilage complex. They may also involve tendons and muscles surrounding the elbow, forearm, wrist, and hand. Injuries may also create entrapment or other forms of distal neuropathies involving the median, ulnar or radial nerves.

Lateral epicondylitis is one of the most prevalent disorders of the arm [5, 6] with a point prevalence of 1–3 % in the adult general population [2, 5, 7] and up to 7.3 % in workers [3, 6, 8]. The incidence of lateral epicondylitis is higher among females and peaks between the ages of 40 and 50 years [9]. Lateral epicondylitis is a self-limiting condition with most cases resolving within three months and up to 89 % of patients report improvement in pain at one year [10, 11]. Recurrence has been reported in up to 8.5 % of cases [10]. Medial epicondylitis is less common with a point prevalence of 0.4 % in adults [5] and 4.3 % in workers [6]. Medial epicondylitis is similarly a self-limiting condition with most cases resolving within 6 to 12 months [12]. Ulnar neuropathy (cubital tunnel syndrome) at the elbow, is the second most common upper extremity peripheral neuropathy [13, 14]. Ulnar neuropathy at the elbow has an annual incidence of 24.7 cases per 100,000 person-years, and is more common in men [14, 15]. Previous work suggests that approximately one half of untreated mild ulnar neuropathy report symptom resolution at one year [16], with resolution of symptoms following non-surgical interventions being inversely proportional to symptom severity [17].

Carpal tunnel syndrome (CTS) is the most common nerve entrapment neuropathy [18–20] with the point prevalence ranging from 1.0 to 3.8 %, and is higher in females than males in the general population [7, 18, 21]. The point prevalence of CTS is higher in workers, ranging from 2.2 to 14.0 % [8, 22–25]. Some CTS cases resolve spontaneously, with initial low impairment severity associated with worsening of symptoms and severe impairment associated with improvement [26]. Similarly, the point prevalence of De Quervain’s tenosynovitis is higher in females (1.3 %) than males (0.5 %) [7], and is higher in workers than the general population [8, 23].

Elbow, forearm, wrist and hand musculoskeletal disorders are associated with pain, functional impairment and decreased productivity [2, 3, 5, 18, 21]. In Canada, lateral epicondylitis is associated with lost productivity and inability to work for up to eight weeks [5, 27]. The estimated annual cost of medical care and lost work time associated with lateral or medial epicondylitis in the United States (US) is $22 billion (USD) [19]. In Alberta, CTS was associated with 800 annual workers’ compensation claims during the time period 1998 to 2002 [28]. Although, average lost work days decreased (151 to 98) between 1998 and 2002 carpal tunnel surgeries increased during this period [28]. With approximately 400,000 carpal tunnel surgeries performed annually at a cost of $2 billion (USD), surgery for CTS is not only the most common but also the most costly upper extremity disorder treatment in the US [21, 29].

Clinicians manage patients’ conditions according to their training, beliefs, preferences and understanding of the evidence. Moreover, clinicians are likely to combine various interventions recognized as multimodal care when managing patients’ conditions. However, randomized control trials (RCTs) commonly examine the effectiveness of single interventions which has limited applicability to clinical practice [30]. Therefore, understanding the effectiveness of multimodal care is important to guide clinical practice and provide the best available care to patients.

To our knowledge, no systematic review has evaluated the effectiveness of multimodal care for the management of musculoskeletal disorders of the elbow, forearm, wrist and hand. The objective of our systematic review was to examine the effectiveness of multimodal care for the management of musculoskeletal disorders of the elbow, forearm, wrist and hand on self-rated recovery, functional recovery or clinical outcomes in adults or children.

## Methods

### Registration of review

This review protocol was registered with the International Prospective Register of Systematic Reviews (PROSPERO) on January 31^st^, 2014 (CRD42014009093).

### Eligibility criteria

#### Population

We included studies of children and adults eighteen years and older diagnosed with musculoskeletal disorders of the elbow, forearm, wrist and hand, including non-specific elbow, forearm, wrist and hand pain, olecranon bursitis, lateral epicondylitis (tennis elbow), medial epicondylitis (golfer’s elbow), ulnar neuropathy (cubital tunnel syndrome), carpal tunnel syndrome, De Quervain’s tenosynovitis, and other musculoskeletal disorders of the elbow, forearm, wrist and hand as informed by available evidence [31]. Study participants who had been clinically diagnosed by a health care professional within their defined scope of practice, were considered for inclusion in our review. Grades I and II sprains or strains of the elbow, forearm, wrist and hand were included in our review. We excluded studies of elbow, forearm, wrist or hand injuries due to major structural pathology (e.g., fractures, dislocations, amputations, open wounds, tears of surrounding structures, osteoarthritis, spinal cord injury or neoplasms). We defined sprains and strains according to the definition proposed by the American Academy of Orthopaedic Surgeons [32–34].

#### Interventions

We defined multimodal care as a treatment approach that includes at least two distinct therapeutic modalities, provided by one or more health care disciplines [35]. Therapeutic modalities included: acupuncture, education, exercise, manual therapy (manipulation, mobilization, traction), passive physical modalities (e.g., heat application, cryotherapy, ultrasound, splints, braces), prescribed medication (e.g., acetaminophen, non-steroidal anti-inflammatory drugs), psychological interventions (e.g., relaxation, biofeedback) and soft-tissue therapies (e.g., massage, muscle energy technique). We excluded studies where the effectiveness of a single intervention could be isolated. For example, if supervised exercise plus an orthosis were compared to an orthosis alone, the effect of supervised exercise could be isolated and the study would not be considered in the analysis of multimodal care.

#### Comparison groups

Studies that compared multimodal care to other interventions, placebo/sham interventions or no intervention were considered.

#### Outcomes

Eligible studies included: 1) self-rated recovery; 2) functional recovery (e.g., return to activities at work or school); 3) disability; 4) pain intensity; 5) health-related quality of life; 6) psychological outcomes (e.g., depression, fear); or 7) adverse events.

#### Study characteristics

Eligible studies met the following criteria: 1) English language; 2) published between January 1^st^, 1990 and March 12^th^, 2015; 3) RCTs, cohort studies, or case–control studies; and 4) included an inception cohort of at least 30 participants per treatment arm with the specified condition for RCTs, or 100 subjects per treatment arm in cohort studies or case–control studies. We considered cohort studies to ensure that all designs that may be used to examine study effectiveness and safety were included in our review. A low risk of bias cohort study may provide a higher level of evidence than a high risk of bias RCT [36–38]. This implies that the investigators of a high-quality cohort study have addressed the potential for confounding by indication. Evidence from well conducted case–control studies is valuable in understanding the risk of adverse events associated with specific treatments and are therefore included in this systematic review. Study exclusion criteria included: 1) letters, editorials, commentaries, unpublished manuscripts, dissertations, government reports, books and book chapters, conference proceedings, meeting abstracts, lectures and addresses, consensus development statements, or guideline statements; 2) pilot studies, cross-sectional studies, case reports, case series, qualitative studies, narrative reviews, systematic reviews, clinical practice guidelines, biomechanical studies, or laboratory studies; or 3) cadaveric or animal studies.

### Data sources and searches

Our search strategy was developed in consultation with a health sciences librarian, and a second librarian reviewed the search for completeness and accuracy using the Peer Review of Electronic Search Strategies (PRESS) Checklist [39, 40]. We searched MEDLINE and EMBASE, considered to be the major biomedical databases, and PsycINFO for psychological literature, through Ovid Technologies, Inc.; CINAHL Plus with Full Text for the nursing and allied health literature through EBSCO host; and the Cochrane Central Register of Controlled Trials, through Ovid Technologies, Inc. for any studies not captured by other databases. Searches were conducted from January 1^st^, 1990 to March 12^th^, 2015.

The search strategy was first developed in MEDLINE and subsequently adapted to the other databases. The search terms included subject headings specific to each database (e.g., MeSH in MEDLINE) [41] and free text words relevant to multimodal care and musculoskeletal disorders of the elbow, forearm, wrist and hand, including sprains and strains grades I-II (Additional file 1). Databases containing the results of the searches were created using EndNote X6 [42].

### Study selection

Eligible studies were selected through a two-phase screening process. In phase one, two randomly paired reviewers independently screened titles and abstracts to determine eligibility. Studies were classified as relevant, possibly relevant or irrelevant. In phase two, the same reviewers independently reviewed manuscripts of possibly relevant studies to make a final determination of eligibility. Reviewers met to resolve disagreements and reach consensus in both phases. We involved a third independent reviewer if consensus could not be reached.

### Quality assessment and data extraction

Independent reviewer pairs critically appraised the internal validity of eligible studies using the Scottish Intercollegiate Guidelines Network (SIGN) criteria for RCTs, cohort studies and case–control studies [43]. The SIGN criteria assist with the evaluation of the impact of selection bias, information bias, and confounding on the results of a study. We did not use a quantitative score or a cutoff point to determine the internal validity of studies [44]. Rather, the SIGN criteria were used to assist reviewers in making an informed overall judgment on the internal validity of studies. This methodology has been previously described [45–48].

Specifically, we critically appraised the following methodological aspects of the studies: 1) clarity of the research question; 2) randomization method; 3) concealment of treatment allocation; 4) blinding of treatment and outcomes; 5) similarity of baseline characteristics between treatment arms; 6) co-intervention/contamination; 7) validity and reliability of outcome measures; 8) attrition; 9) intention to treat analysis; and 10) comparability of results across study sites (where applicable). A study was considered to have a high risk of bias if reviewers considered that the study’s internal validity was compromised as a result of biases and methodological flaws, including: a) a RCT with an inadequate randomization method and/or without clear concealment of treatment allocation, and with non-random distribution of baseline characteristics which were not controlled for in the analysis; b) unexplained high or differential attrition rates; c) absence of an intention to treat analysis in order to provide a conservative estimate of therapeutic effect; or, d) outcome measures without established validity and reliability. Moreover, inadequate blinding, an imbalance of co-interventions and lack of comparability across treatment sites (if applicable) were considered as additional limitations. All reviewers were trained in the evaluation of studies using the SIGN criteria. Consensus between reviewers was reached through discussion. An independent third reviewer was used to resolve disagreements if consensus could not be reached. We contacted authors when additional information was needed to complete the critical appraisal. Studies with a low risk of bias were included in our evidence synthesis [49].

The lead author extracted data from studies with a low risk of bias into evidence tables. A second reviewer independently checked the extracted data. Meta-analysis was not performed due to heterogeneity of studies with low risk of bias.

### Data synthesis and analysis

A qualitative synthesis of the low risk of bias studies was performed according to principles of best evidence synthesis [49]. We stratified our synthesis according to disorder type and duration [i.e., recent (<3 months), persistent (≥3 months)]. We further stratified the multimodal programs of care according to their effectiveness to determine the components of intervention that are associated with superior outcomes: 1) superior (associated with a minimal clinically important difference (MCID) compared to its comparator) [50]; 2) equivalent (no clinically important differences between groups); and 3) inferior (associated with worse outcomes than its comparator). The following MCID thresholds were employed: Carpal Tunnel Syndrome Assessment Questionnaire (CTSAQ)-Symptom Severity Scale (SSS) 0.16/5 [51], and Functional Status Scale (FSS) 0.47/5 [51]; 14/100 mm on the visual analogue scale (VAS) [52]; 2/10 difference on the Numeric Rating Scale (NRS) [53]; 11/100 or 37 % of baseline score for the Patient-rated Tennis Elbow Evaluation (PRTEE) [54]; and 6.5 kg (19.5 %) for grip strength [55]. We estimated the intensity of care by computing the mean number of visits and treatment duration for superior outcomes associated with each disorder type.

### Statistical analyses

The inter-rater agreement for article screening was computed using the kappa coefficient (*ĸ*) and 95 % confidence intervals (*CI*) [56, 57]. The percentage agreement for critical appraisal was calculated for low and high risk of bias studies. Similarly, we computed the difference in mean change between groups and its 95 % *CI* to quantify effect sizes. The computation of the 95 % *CI* for the difference in mean change assumed that the pre- and post-intervention outcomes were highly correlated (*r* = 0.8) [58, 59].

### Reporting

The systematic review was organized and reported based on the Preferred Reporting Items for Systematic Reviews and Meta-Analyses (PRISMA) statement [60].

## Results

### Study selection

**Fig. 1:**
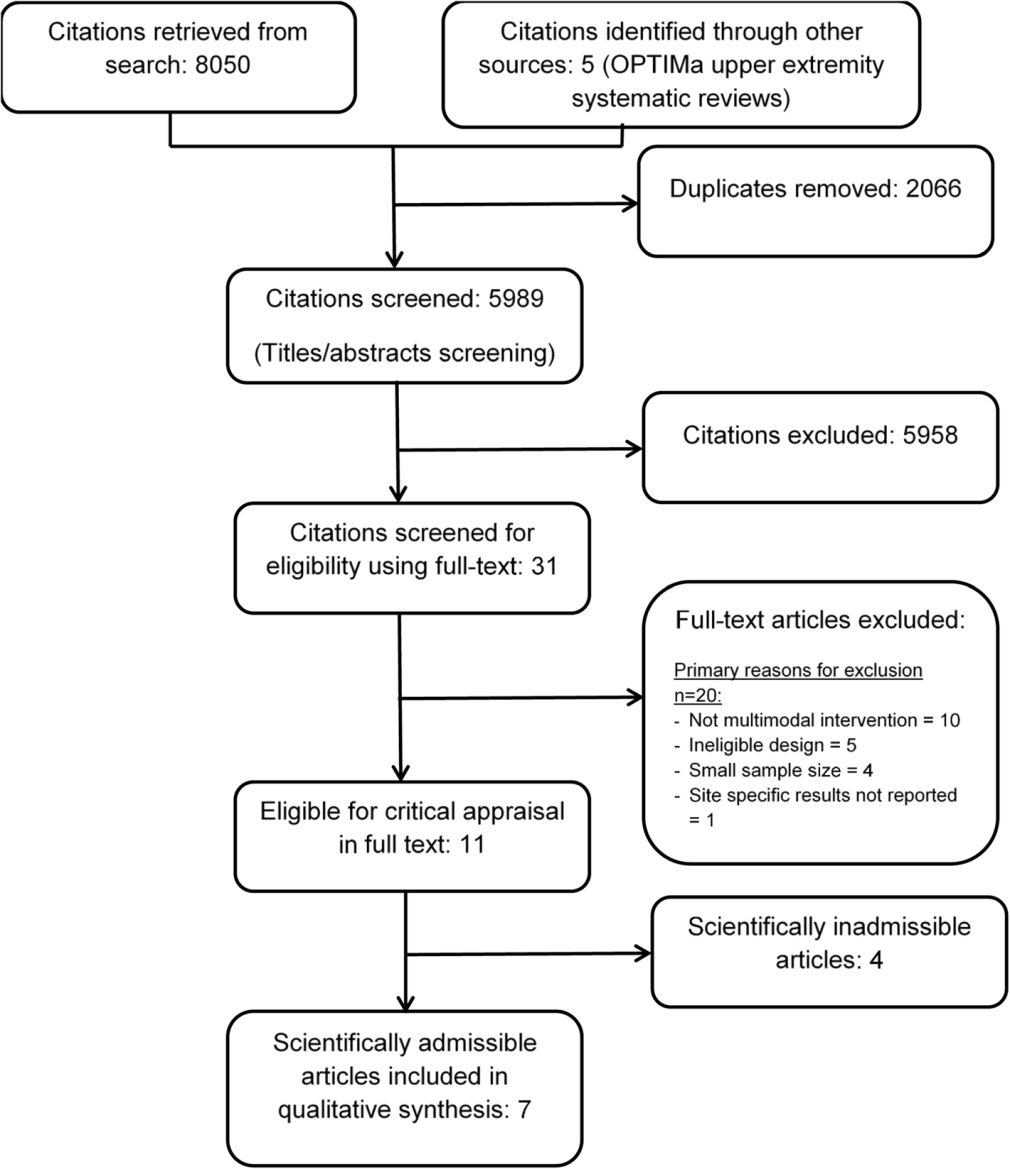
Identification and selection of articles

Our search yielded 8050 articles. We removed 2066 duplicates and screened 5989 articles (Fig. 1). Of those, 11 articles (reporting on ten studies) were eligible for critical appraisal and seven articles (six studies) had a low risk of bias and were included in our synthesis [61–67]. During critical appraisal of articles we contacted the authors of five studies for further information and clarification [61, 64, 65, 68, 69] and three responded [61, 65, 69].

The inter-rater agreement for the screening of articles was *ĸ* = 0.84 (95 % CI 0.67; 1.00). The percentage agreement for the critical appraisal of articles was 90.9 % (10/11 articles). For the study where reviewers disagreed, consensus was reached through discussion. The two reviewers met and reviewed each methodological criterion using a standardized approach (SIGN criteria), ensuring that a comprehensive analysis of the potential sources of selection and information bias, as well as confounding, and their impact on the study were discussed. A final consensus judgment on the quality of the study was then determined by both reviewers.

### Study characteristics

All six studies (seven articles) with low risk of bias were RCTs. One study addressed CTS [61], and five studies (six articles) examined lateral epicondylitis [62–67].

There was a range of interventions reported in the multimodal programs of care including: education, exercise, manual therapy (manipulation), soft-tissue therapy (deep friction massage); passive physical modalities (ultrasound, splints), prescribed medication and usual physician care (Tables 1 and 2). Exercise (5/5), education (3/5) and soft tissue therapy (3/5) were the most common interventions included in a multimodal program of care for the management of lateral epicondylitis. Two health care disciplines were identified in the delivery of multimodal programs of care for CTS (hand therapist, general practitioner), and three for lateral epicondylitis (physical therapist, general practitioner, ergonomist). Two studies, one for the management of CTS [61] and one for lateral epicondylitis included more than one health care discipline to deliver a single multimodal care intervention [64].

**Table 1:** Combinations of Interventions in Multimodal Care for Lateral Epicondylitis Reported in Scientifically Admissible Studies, 1990–2015^a,b^

				Education	Exercise	Manual Therapy			Soft Tissue Therapy	Acupuncture	Passive Modalities			Medication	Usual Care
Author, year	Treatment provider	Number of visits	Treatment period (weeks)			Manipulation	Mobilisation	Traction			Ultrasound	Splint	Heat/Cold		
Bisset [62, 63]	PT^c^	8	6	✓	✓	✓									
	GP^e^	2	6	✓										✓	
	GP^d^	1	6	✓											
Smidt [66]	PT^c^	9	6		✓				✓		✓				
	GP^e^	3	6											✓	
	GP^d^	1	6											✓	✓
Haahr [64]	GP, Ergonomist^d^	1	UK	✓	✓									✓	
	GP^d^	1	UK												✓
Nagrale [65]	PT^c^	12	4	✓		✓			✓						
	PT^e^	12	4	✓	✓						✓				
Struijs [67]	PT^d^	9	6		✓				✓		✓				
	PT^d^	1	6									✓			
	PT^d^	9	6		✓				✓		✓	✓			

^a^Empty cells indicate that the intervention component was not provided in the treatment arm

^b^Table includes only modalities reported in scientifically admissible studies

Acronyms: *GP* general practitioner, *PT* physical therapist, *UK* unknown

^c^Superior multimodal programs of care; ^d^Equivalent multimodal programs of care; ^e^Inferior multimodal programs of care

**Table 2:** Combinations of Interventions in Multimodal Care for Carpal Tunnel Syndrome Reported in Scientifically Admissible Randomized Controlled Trials, 1990–2015^a,b^

				Education	Exercise	Manual Therapy			Soft Tissue Therapy	Acupuncture	Passive Modalities		Surgery
Author, Year	Treatment Provider	Number of Visits	Treatment Period (weeks)			Manipulation	Mobilisation	Traction			Ultrasound	Splint	
Jarvik [61]	GP, HT^c^	30	12	✓	✓						✓	✓	
	Surgeon, HT^d^	UK	UK		✓								✓

^a^Empty cells indicate that the intervention component was not provided in the treatment arm

^b^Table includes only modalities reported in scientifically admissible studies

Acronyms: *GP* general practitioner, *HT* hand therapist

^c^Superior multimodal programs of care; ^d^Inferior multimodal programs of care

### Risk of bias within studies

All RCTs with a low risk of bias used appropriate randomization procedures, valid and reliable outcome measures and performed an intention-to-treat analysis (Table 3) [61–67]. The follow-up rate was above 85 % for all but one RCT [61–63, 65–67]. The study by Haahr et al. reported follow-up rates above 75 % [64]. However, the studies with a low risk of bias had some limitations including: 1) differences in baseline characteristics (2/6) [64, 66]; and 2) no description of co-interventions (3/6) [62–66].

**Table 3:** Risk of Bias for Accepted Randomized Controlled Trials based on the Scottish Intercollegiate Guidelines Network (SIGN) Criteria [43]

Author, Year	Research Question	Randomization	Concealment	Blinding	Similarity at baseline	Similarities between arms	Outcome measurement	Percent drop-out	Intention to treat	Multiple sites
Bisset et al., 2006, 2009 [62, 63]	Y	Y	Y	Y	Y	N	Y	6 Weeks: Multimodal Care: 5 % Corticosteroid Injection: 0 % Reassurance and advice: 10 % 52 Weeks: Multimodal Care: 5 % Corticosteroid Injection: 0 % Reassurance and advice: 7 %	Y	CS
Haahr et al., 2003 [64]	Y	Y	Y	Y	N	N	Y	3 Months: Multimodal GP/Ergonomist: 16 % GP: 14 % 6 Months: Multimodal GP/Ergonomist: 19 % GP: 16 % 12 Months: Multimodal GP/Ergonomist: 22 % GP: 18 %	Y	CS
Jarvik et al., 2009 [61]	Y	Y	Y	Y	Y	Y	Y	12 Months: Multimodal Care: 11.9 % Surgery: 14.0 %	Y	Y
Nagrale et al., 2009 [65]	Y	Y	Y	Y	Y	N	Y	No drop outs	NA	CS
Smidt et al., 2002 [66]	Y	Y	Y	CS	N	N	Y	Multimodal PT: no drop outs Reassurance and advice: no drop outs Corticosteroid injection: 12 weeks: 1.6 % 26 weeks: 1.6 %	Y	CS
Struijs et al., 2004 [67]	Y	Y	CS	Y	Y	Y	Y	6 Weeks: Multimodal PT: 3.6 % Brace: 1.5 % Multimodal PT + Brace: 1.8 % 26 Weeks: Multimodal PT: 3.6 % Brace: 5.9 % Multimodal PT + Brace: 3.6 % 52 Weeks: Multimodal PT: 5.4 % Brace: 5.4 % Multimodal PT + Brace: 3.6 %	Y	NA

Acronyms: *CS* cannot say, *GP* general practitioner, *N* no, *NA* not applicable, *PT* physical therapy, *Y* yes

Four studies had high risk of bias [69–72]. The methodological weaknesses of the three RCTs include: inadequate description of randomization (3/3) [70–72]; blinding of treatment and outcome assessment not described (3/3) [70–72]; no information regarding co-interventions (3/3) [70–72]; valid and reliable outcome measures not used (2/3) [70, 72]; and intention to treat analyses not used (3/3). The fourth study was a cohort study which did not adequately describe the source population; baseline characteristics of the sample were not described; and potential confounders were not adjusted for in the analysis [69].

### Summary of evidence

#### Carpal tunnel syndrome of persistent duration

Evidence from one RCT suggests that multimodal care provided by a physician and a hand therapist is less effective than decompression surgery and hand therapy for the management of carpal tunnel syndrome (Table 4) [61]. In their trial Jarvik et al. [61] randomized participants to: 1) multimodal care that included a combination of non-steroidal anti-inflammatory drugs, an education booklet, exercise, stretching, splint (night and as tolerated by day), ultrasound and home/workplace modifications; 2) open or endoscopic decompression surgery followed by hand therapy (median nerve and tendon gliding exercises). Compared with multimodal care, surgery plus hand therapy led to statistically but non-clinically important improvement in functional status (Boston Carpal Tunnel Syndrome Questionnaire) at six (mean change difference -0.46/5 (95 % *CI* -0.72; -0.20)) and twelve months (mean change difference -0.40/5 (95 % *CI* -0.70; -0.11)). Surgery was associated with statistically and clinically important differences in the secondary outcome-severity of symptoms (Boston Carpal Tunnel Syndrome Questionnaire) at six (mean change difference -0.42/5 (95 % *CI* -0.77; -0.07)) and twelve months (mean change difference -0.34/5 (95 % *CI* -0.65; -0.02)). The authors reported no differences in other secondary outcomes.

**Table 4:** Evidence table for accepted randomized control trials on multimodal care and carpal tunnel syndrome

Author(s), Year	Subjects and Setting; Number (n) Enrolled	Interventions; Number (n) of Subjects	Comparisons; Number (n) of Subjects	Follow-up	Outcomes	Key Findings
Jarvik et al., 2009 [61]	Participants (≥18 y.o.) recruited from Washington State and New Hampshire. Case definition: symptoms ≥2 weeks in at least 2 digits including thumb, index, ring finger; CTS on hand pain diagram, electrodiagnostic testing (motor latency, ulnar sensory difference, radial sensory difference); night pain waking; positive flick test. (*n* = 116).	Multimodal Care by physician and hand therapist: NSAIDS (ibuprofen 200 mg/3x /day), opioid, corticosteroid; hand therapy (6 visits/6 weeks): educational booklet, exercises, stretching, tendon gliding, wrist splint and work/activity modifications; ultrasound if no improvement 6 weeks after randomization (maximum 12 15-min sessions; 2–4 per week/6 weeks): 1Mhz, 1.0 W/cm^2^ in 1:4 pulsed mode. (*n* = 59)	Surgery: open or endoscopic decompression, followed by hand therapy (median nerve and tendon gliding exercises). (*n* = 57)	6 and 12 months	Primary Outcome: Function (CTSAQ Functional Status Scale) Secondary Outcomes: Symptom severity (CTSAQ Symptom Severity Scale); hand/wrist pain intensity (NRS 0–10); hand/wrist pain interference (NRS 0–10); work days lost (0–28); limited activity days; general health-related quality of life (SF-36 0–100). Successful outcome: ≥0.5 points improvement from baseline CTSAQ function; ≥0.5 points CTSAQ symptom severity; and a score of 0 or 1 on hand/ wrist pain interference with work or housework Adverse events.	^*^Difference in Mean Change Score (Multimodal Care-Surgery) CTSAQ Function 6 months: -0.46 (95 % *CI* -0.72; -0.20) 12 months: -0.40 (95 % *CI* -0.70; -0.11) CTSAQ Symptom Severity 6 months: -0.42 (95 % *CI* -0.77; -0.07) 12 months: -0.34 (95 % *CI* -0.65; -0.02) There were no clinically or statistically differences between groups in days of reduced work/housework, work days lost, pain intensity, pain interference or SF-36 at any follow-up point. Successful Outcome^a^ 6 months: Multimodal Care: *RR* 0.51 (95 % *CI* 0.25; 1.05) 12 months: Multimodal Care: *RR* 0.62 (95 % *CI* 0.35; 1.08) Adverse Events: No clinically important adverse events; no surgical complications.

^a^Calculated by OPTIMa team (Follman (1992); Abrams (2005))

*ANCOVA adjusted for baseline value of the outcome measure and treatment site

Acronyms: *CI* Confidence Interval, *CTSAQ* Carpal Tunnel Syndrome Assessment Questionnaire, *NRS* numeric rating scale, *SF-36* short form 36, *y.o*. years old

#### Lateral epicondylitis of persistent duration

##### Multimodal care compared to corticosteroid injection

Evidence from one RCT suggests that multimodal care by a physical therapist provides long term benefits compared to corticosteroid injection plus education by a physician, and offers similar outcomes to reassurance and advice by a physician (Table 5) [62, 63]. In their RCT, Bisset et al. [62, 63] randomized participants to: 1) multimodal care (manipulation, clinic and home based exercise) provided in eight sessions over six weeks; 2) corticosteroid injection of the painful elbow joint and advice to return to normal activities (a second injection was offered after two weeks if necessary); or 3) reassurance and advice on self-management (activity modification; analgesic drugs, heat, cold or braces as needed). All participants received an information booklet covering the disease process, self-management, and ergonomics. Participants who received the corticosteroid injection demonstrated greater improvement in pain (mean change difference -15.8/100 (99 % *CI* -26.4; -5.1)) and were more likely to report self-perceived improvement (*RR* 0.79 (99 % *CI* 0.63; 0.99)) at six weeks post-intervention; however, those in the multimodal care group were more likely to report self-perceived improvement (*RR* 1.32 (99 % *CI* 1.09; 1.59)) and greater pain reduction (mean change difference 14.5/100 (99 % *CI* 4.2; 24.8)) at 52 weeks. Multimodal care participants were less likely to experience a recurrence than the corticosteroid injection group (*RR* 0.11 (95 % *CI* 0.05; 0.25)). Participants receiving multimodal care reported clinically important improvement in pain (mean change difference 15.6/100 (99 % *CI* 7.3; 22.8)) at six weeks but not 52 weeks compared to the reassurance and advice group. Those in the multimodal care group were more likely to report self-perceived improvement than those in the reassurance and advice group (*RR* 2.60 (99 % *CI* 1.63; 4.15)) at 6 weeks, but not at 52 weeks. However, the proportion of patients reporting self-perceived improvement is higher in the multimodal care group at each follow-up point compared to the reassurance and advice group. It is not until 26 weeks that the reassurance and advice group report a similar proportion of global improvement as the multimodal care group reported at six weeks. Although the probability of self-perceived improvement is similar at 52 weeks, the multimodal care group reports positive sustained outcomes at a much earlier time point compared to the reassurance and advice group.

**Table 5:** Evidence table for accepted randomized controlled trials on multimodal care and lateral epicondylitis

Author(s), Year	Subjects and Setting; Number (n) Enrolled	Interventions; Number (n) of Subjects	Comparisons; Number (n) of Subjects	Follow-up	Outcomes	Key Findings
Bisset et al., 2006, 2009 [62, 63]	Participants (18–65 y.o.) from Brisbane, Australia. Case definition: lateral elbow pain increased with palpation of the lateral epicondyle, gripping, resisted wrist or second or third finger extension of >6 weeks duration. (*n* = 198)	Multimodal care by a PT (8 visits/6 weeks): elbow manipulation, exercise (supervised and home-based), self-manipulation, educational booklet (disease process, self management advice, ergonomics). (*n* = 66)	Corticosteroid injection by a GP (1 ml 1 % lidocaine with 10 mg triamcinolone acetonide in 1 ml); 1 injection at painful points and second injection after two weeks if necessary; advice to return gradually to normal activities; educational booklet (disease process, self management advice, ergonomics) (*n* = 65) Reassurance and Advice: reassurance (ADL modifications, analgesic drugs, heat, cold, braces), educational booklet (disease process, self management advice, ergonomics) (*n* = 67)	6, 12, 26 and 52 weeks	Primary Outcome: Global improvement (6 point Likert Scale); success = “completely recovered” or “much improved”; recurrence (“successful” to “unsuccessful”); pain-free grip force (digital grip dynamometer, affected side/unaffected side x 100) Secondary Outcome: pain severity (VAS 0–100 mm); elbow disability (Pain Free Function Questionnaire (PFFQ)); Sensorimotor function: SRT(ms); RT1(ms); RT2(ms); S1(cm/s); S2(cm/s) Adverse events.	Relative Risk (Multimodal Care vs. Corticosteroid Injection):^a^ Success 6 weeks: *RR* 0.79 (95 % *CI* 0.63; 0.99) 12 weeks: *RR* 1.53 (95 % *CI* 1.11; 2.10) 26 weeks: *RR* 1.73 (95 % *CI* 1.28; 2.34) 52 weeks: *RR* 1.32 (95 % *CI* 1.09; 1.59) Recurrence After 6 weeks: *RR* 0.11 (95 % *CI* 0.05; 0.25) Difference in Mean Change from Baseline: (Multimodal Care - Corticosteroid Injection^b^) Pain-free Grip Force 6 weeks:--17.4 (99 % *CI* -22.4; -12.4) 12 weeks: 13.1 (99 % *CI* 7.6; 18.6) 26 weeks: 28.2 (99 % *CI* 21.6; 34.8) 52 weeks: 12.3 (99 % *CI* 6.7; 17.9) Pain Severity 6 weeks: -13.4 (99 % *CI* -18.8; -8.0) 12 weeks: 19.4 (99 % *CI* 13.6; 25.2) 26 weeks: 20.0 (99 % *CI* 14.6; 25.4) 52 weeks: 18.2 (99 % *CI* 12.6; 23.8) PFFQ 6 weeks: -20.3 (99 % *CI* -26.9; -13.6) 12 weeks: 12.4 (99 % *CI* 5.1; 19.7) 26 weeks: 21.4 (99 % *CI* 15.1; 27.6) 52 weeks: 18.8 (99 % *CI* 11.9; 25.7) Sensorimotor Function There were no clinical or statistical differences between groups in SRT, RT1, RT2, S1 or S2 at any follow-up point. Relative Risk Reduction (Multimodal Care vs. Reassurance and Advice):^a^ Success 6 weeks: *RR* 2.60 (99 % *CI* 1.63; 4.15) 12 weeks: *RR* 1.31 (99 % *CI* 0.98; 1.73) 26 weeks: *RR* 1.08 (99 % *CI* 0.88; 1.32) 52 weeks: *RR* 1.07 (99 % *CI* 0.93; 1.22) Recurrence 6 weeks: *RR* 0.85 (95 % *CI* 0.27; 2.65) Difference in Mean Change from Baseline (Multimodal care – Reassurance and Advice^b^): Pain-free Grip Force 6 weeks: 24.0 (99 % *CI* 19.0; 29.0) 12 weeks: 14.3 (99 % *CI* 9.5; 19.1) 26 weeks: 15.4 (99 % *CI* 9.9; 20.9) 52 weeks: 10.0 (99 % *CI* 4.4; 15.6) Pain Severity 6 weeks: 12.2 (99 % *CI* 7.3; 17.1) 12 weeks: 5.8 (99 % *CI* 0.8; 10.8) 26 weeks: 4.9 (99 % *CI* -0.5; 10.3) 52 weeks 1.4 (99 % *CI* -4.2; 7.0) PFFQ 6 weeks: 15.7 (99 % *CI* 9.9; 21.5) 12 weeks: 17.4 (99 % CI 11.2; 23.6) 26 weeks: 5.0 (99 % *CI* -1.1; 11.1) 52 weeks: 10.4 (99 % *CI* 4.1; 16.7) Sensorimotor Function There were no clinical or statistical differences between groups in SRT, RT1, RT2, S1 or S2 at any follow-up point. Adverse Events Minor: pain following treatment, loss of skin pigment; subcutaneous tissue atrophy. Multimodal Care: 10.6 %; Corticosteroid Injection: 20.0 %; Wait and See: 0.0 %.
Haahr et al., 2003 [64]	Adults (18–66 y.o.) with lateral epicondylitis consulting with GP in Ringkjoebing County, Denmark. Case definition: new episode (<1 year) of lateral epicondylitis (i.e., indirect tenderness at or within 2 cm from lateral humeral epicondyle on resisted extension of wrist and/or third finger (*n* = 289).	Multimodal Intervention by GP and ergonomist: reassurance and advice (against complete rest, stay active, avoid aggravating activities, adjust work conditions) by GP. Graded exercises by ergonomist. OTC analgesics, (*n* = 148)	Usual care provided by GP. (*n* = 141)	1 year	Primary Outcome: Self-reported overall development of condition (5 point Likert; ‘much better’ to ‘much worse’); 50 % reduction in combined pain and function score Secondary Outcome: sickness absence, ceased or changed job, start education or rehabilitation activity, labour compensation claim.	Perceived unchanged or worse overall development^c^: Control: *OR* 1.0 Multimodal Care: *OR* 1.0 (95 % *CI* 0.4; 2.3)
Nagrale et al., 2009 [65]	Outpatient clinic, Wardha, Maharashtra, India (30–60 y.o.). Case definition: tenderness to palpation over the lateral humeral epicondyle, pain with gripping, passive wrist flexion with elbow extension and resisted wrist extension lasting ≥1 month (*n* = 60)	Cyriax physiotherapy (deep transverse friction massage plus Mill’s manipulation), education (ergonomics, activity modification) (3/week; 4 weeks). (*n* = 30)	Phonophoresis (5 min) over lateral epicondyle (Voveran Emulgel frequency 1 MHz, 0.8 W/cm^2^ intensity), supervised exercise, education (ergonomics, activity modification) (3/week; 4 weeks) (*n* = 30)	4 and 8 weeks	Primary Outcome: pain severity (VAS 0–10 cm); pain-free grip strength (dynamometer, pounds); Tennis Elbow Function Scale (TEFS) (0–40)	Cyriax Physiotherapy – Phonophoresis + Exercise Difference in Mean Change Score ^a,d^ Pain severity 4 weeks: 1.8 8 weeks: 2.5 Pain-free Grip Strength 4 weeks: 12.4 8 weeks: 14.5 TEFS 4 weeks: 7.7 8 weeks: 8.9
Smidt et al., 2002 [66]	Primary care setting (85 family doctors), referred to 5 research centres, Netherlands (18–70 y.o.). Case definition: lateral epicondylitis ≥6 weeks duration (*n* = 185)	Multimodal Care: provided by a PT (9visits/6 weeks): pulsed ultrasound, massage, exercise; home exercise equipment and instruction booklet.(*n* = 64)	Corticosteroid Injection (1 mL of 10 mg/mL triamcinolone cetonide + 1 mL 2 % lignocaine) at each tender spot; maximum 3 injections in 6 weeks, avoid pain-provoking activities provided by family doctor (*n* = 62) Reassurance and Advice: 1 visit with family doctor in 6 weeks. Advice (pain provoking activities, ergonomic), paracetamol or NSAIDs if necessary, await spontaneous improvement (*n* = 59)	6, 12, 26 and 52 weeks	Primary Outcome: Global improvement (“completely recovered“to “much worse”); success = “completely recovered” or “much improved”; severity of main complaint (NRS 0–10); pain during day (NRS 0–10); inconvenience (NRS 0–10); functional disability (modified pain-free function questionnaire, 0–40); PT rated overall severity (0–10). Secondary Outcomes: pain-free grip strength (kg); maximum grip strength (kg); pressure pain threshold; satisfaction with intervention. All scales transformed to 0–100. Adverse events.	Multimodal Care – Reassurance and Advice Difference in Mean Change Score ^a^ Success rate 6 weeks: *RR* 1.46 (95 % *CI* 0.93; 2.29) 52 weeks: *RR* 1.09 (95 % *CI* 0.95; 1.25) Multimodal Care - Corticosteroid injection Success rate 6 weeks: *RR* 0.51 (95 % *CI* 0.39; 0.67) 52 weeks: *RR* 1.31 (95 % *CI* 1.09; 1.57) Adverse events: increased pain; radiating pain; facial flush; skin irritation; red swollen elbow; skin colour change; other minor or temporary adverse reactions. Multimodal: 64 %; Corticosteroid Injection:58 %; Reassurance and Advice: 17 %
Struijs et al., 2004 [73]	Patients referred from GP and primary care PT to outpatient clinic, the Netherlands. Case definition: pain aggravated by pressure on lateral epicondyle and resisted wrist dorsiflexion (≥6 weeks duration) (*n* = 180)	Multimodal Care by PT (9 visits/6 weeks): pulsed ultrasound, friction massage, strengthening and stretching exercise, home exercise with diary. (*n* = 56)	Brace: provided by PT (1 visit): Epipoint elbow brace worn over common extensor tendon. (*n* = 68) Combination Group: Multimodal Care plus Brace intervention. (*n* = 56)	6, 26 and 52 weeks	Primary Outcome: Global improvement (6 point Likert Scale) (“completely recovered“to “much worse”); success = “completely recovered” or “much improved”; severity of complaints (0–11 NRS); pain intensity of most severe complaint (0–11 NRS); modified PFFQ (10 item, 0–4) Secondary Outcome: Inconvenience during daily activities (0–10); pain-free grip strength (kg); maximum grip strength (kg); pressure pain threshold at lateral epicondyle (kg/cm^2^); satisfaction with treatment (0–10). All outcomes transformed to 100 point scale.	Multimodal Care – Brace Success rate 6 weeks: *RR* 1.22 (95 % *CI* 0.9; 1.7) 26 weeks: *RR* 0.89 (95 % *CI* 0.5; 1.6) 52 weeks: *RR* 1.26 (95 % *CI* 0.5; 3.3) Difference in Mean Change Score Severity of Complaints 6 weeks: 5 (95 % *CI* -2; 12) 52 weeks: -1 (95 % *CI* -10; 5) Pain Intensity 6 weeks: 13 (95 % *CI* 3; 21) 26 weeks: -1 (95 % *CI* -12; 10) 52 weeks: 0 (95 % *CI* -10; 11) PFFQ 6 weeks: 7 (95 % *CI* 1; 12) 26 weeks: 0 (95 % *CI* -6; 7) 52 weeks: -3 (95 % *CI* -9; 3) There were no clinical or statistical differences between groups in inconvenience during daily activities, pain-free grip strength, maximum grip strength or pressure pain threshold at any follow-up point. Satisfaction 6 weeks: 9 (95 % *CI* 1, 18) Multimodal Care – Combination Success rate 6 weeks: *RR* 0.90 (95 % *CI* 0.6; 1.3) 26 weeks: *RR* 1.31 (95 % *CI* 0.7; 2.4) 52 weeks: *RR* 0.87 (95 % *CI* 0.3; 2.4) Difference in Mean Change Score Severity of Complaints 6 weeks: -6 (95 % *CI* -12; 1) 52 weeks: -3 (95 % *CI* -11; 4) Pain Intensity 6 weeks: 7 (95 % *CI* -4; 17) 26 weeks: -4 (95 % CI -14; 7) 52 weeks: 2 (95 % CI -8; 13) PFFQ 6 weeks: -2 (95 % *CI* -8; 4) 26 weeks: -6 (95 % *CI* -12; 1) 52 weeks: -5 (95 % *CI* -12; 1) There were no clinical or statistical differences between groups in inconvenience during daily activities, pain-free grip strength, maximum grip strength or satisfaction at any follow-up point. Pressure pain threshold 6 weeks: −13 (95 % *CI −*25; −1) Combination--Brace Success rate 6 weeks: *RR* 1.11 (95 % *CI* 0.8; 1.5) 26 weeks: *RR* 1.17 (95 % *CI* 0.6; 2.2) 52 weeks: *RR* 1.10 (95 % *CI* 0.4; 2.8) Difference in Mean Change Score Severity of Complaints 6 weeks: 11 (95 % *CI* 6; 18) 52 weeks: 1 (95 % *CI −*6; 8) Pain Intensity 6 weeks: 6 (95 % *CI −*15; 4) 26 weeks: 5 (95 % *CI −*7; 17) 52 weeks: −2 (95 % *CI −*12; 8) PFFQ 6 weeks: 9 (95 % *CI* 2; 15) 26 weeks: 6 (95 % *CI −*1; 13) 52 weeks: 2 (95 % *CI −*5; 9) There were no clinical or statistical differences between groups in inconvenience during daily activities, pain-free grip strength, maximum grip strength or pressure pain threshold at any follow-up point. Satisfaction 6 weeks: 11 (95 % *CI* 3; 19)

^a^Calculated by OPTIMa team [58, 59]

^b^Adjusted for baseline value of outcome measure and demographic characteristics

^c^:Adjusted for gender, age, education, BMI, physical activity, physical strain at work, social support at work, pain in shoulder or forearm/hand past 3 months, baseline distress, baseline pain, tennis elbow on dominant side

^d^:*P* < 0.05, CI not provided

Acronyms: *CI* Confidence Interval, *CTSAQ* Carpal Tunnel Syndrome Assessment Questionnaire, *GP* general practitioner, *GRC* Global Rating of Change, *OR* Odds Ratio, *PFFQ* Pain Free Function Questionnaire, *PRTEE* Patient-rated Tennis Elbow Evaluation, *PT* physiotherapy, *RR* Relative Risk, *RT1* 1-choice reaction time, *RT2* 2-choice reaction time, *S1* 1-choice speed of movement, *S2* 2-choice speed of movement, *SRT* Simple Reaction Time, *VAS* Visual Analogue Scale, *y.o.* years old

Evidence from a second RCT suggests that multimodal care by a physical therapist provides long term benefits compared to corticosteroid injection and advice by a general practitioner (GP), but offers similar outcomes to reassurance and advice (Table 5) [66]. In their RCT, Smidt et al. [66] randomized participants to: 1) multimodal care (pulsed ultrasound, deep friction massage and an exercise program) provided in nine sessions over six weeks; 2) corticosteroid injection delivered to tender spots (maximum three injections over six weeks) and advice to avoid pain provoking activities; or 3) reassurance and advice (reassurance, avoid activities that provoke pain; ergonomic advice; paracetamol or non-steroidal anti-inflammatory drugs). Participants allocated to multimodal care were less likely to report self-perceived improvement at six weeks (short term) (*RR* 0.51 (95 % *CI* 0.39; 0.67)), but more likely to report improvement at 52 weeks (*RR* 1.31 (95 % *CI* 1.09; 1.57)) than those who received the corticosteroid injection. No differences were found between participants allocated to multimodal care and those in the reassurance and advice group.

##### Multimodal care compared to other interventions

Evidence from one RCT suggests that multimodal care provided by a general practitioner and ergonomist leads to similar outcomes as usual care offered by a GP for the management of lateral epicondylitis (Table 5) [64]. Haahr et al. [64] randomized participants to multimodal care (advice against complete rest, stay active, avoid activities which exaggerate pain, graded exercise, analgesics, “elbow bandages”) by a GP and ergonomist or usual GP care. The authors found no difference between groups in perceived change of condition (*OR* 1.0 (95 % *CI* 0.4; 2.3)) at one year follow-up.

Evidence from one RCT suggests that multimodal care including Cyriax physiotherapy may offer greater benefit than multimodal care including phonophoresis for the management of lateral epicondylitis (Table 5) [65]. In their RCT, Nagrale et al. [65] randomized participants to: 1) Cyriax physiotherapy (deep transverse friction massage, Mill’s manipulation); or 2) phonophoresis, with Voveran Emulgel, over the lateral epicondyle, and supervised exercise (static stretching, eccentric strengthening). All participants received education (ergonomics, activity modification) to avoid provoking symptoms and remain active, each provided in 12 visits over four weeks. Participants who received Cyriax physiotherapy reported statistically and clinically important improvement in pain severity at four (mean change difference 1.8) and six weeks (mean change difference 2.5) (95 % *CI* data not provided; *p* < 0.05). The Cyriax physiotherapy group reported greater improvement in grip strength and the Tennis Elbow Function Scale at both time points (*p* < 0.05), however the clinical importance of these outcomes are not known.

Evidence from one RCT suggests that multimodal care and an elbow brace in combination or as separate independent treatments provided by a physical therapist had similar outcomes (Table 5) [67]. In their RCT, Struijs et al. [67] randomized participants to: 1) multimodal care (pulsed ultrasound, friction massage, clinic and home exercise program); 2) elbow brace worn continuously for six weeks over the common extensor tendon; or 3) a combination of the above. There were statistically significant short-term differences (six weeks) favouring multimodal care (pain intensity, Pain Free Function Questionnaire (PFFQ)) and combined therapy (severity of complaints, PFFQ) over brace alone. However, these differences were not clinically important. No statistically significant or clinically important differences were found at 26 or 52 weeks.

#### Components of effective multimodal programs of care

The multimodal programs of care that benefited lateral epicondylitis included education, exercise (strengthening, stretching, occupational exercises), manual therapy (manipulation) and soft tissue therapy (deep friction massage) (Table 5) [62, 63, 65, 66]. An average of 5 visits (range 3 to 12) offered over five weeks (range 4 to 6 weeks) was associated with superior outcomes for the management of lateral epicondylitis.

#### Adverse events

Three studies reported on adverse events and all were mild and transient [61–63, 66]. Jarvik et al. indicated that there were no clinically important adverse events and there were no surgical complications [61]. Bisset et al. reported that the greatest number of adverse events were associated with corticosteroid injection (20.0 %) compared to multimodal care (10.6 %) or reassurance and advice (0.0 %) [62, 63]. The most common adverse event was pain after treatment (19/20 events). However, Smidt et al. reported more adverse events for the multimodal care group (64 %) compared to corticosteroid injection (58 %) or reassurance and advice (17 %) [66]. The most frequently reported adverse event in all groups was radiating pain to the forearm or upper arm, followed by increased pain lasting greater than one day, and increased pain lasting less than one day.

## Discussion

### Summary of evidence

We conducted a systematic review to evaluate the effectiveness of multimodal care for the management of musculoskeletal disorders of the elbow, forearm, wrist and hand. Overall, we identified one multimodal program of care for the management of persistent CTS. The best evidence suggests that multimodal care (NSAIDs, education booklet, exercise, stretching, splint, ultrasound, home/workplace modifications) was not as effective as decompression surgery and hand therapy for reduction of symptom severity [61].

Our review suggests that there may be a role for multimodal care in the management of patients with persistent lateral epicondylitis. Specifically, we found that multimodal care was more effective in the long-term, than corticosteroid injection but was equally effective to reassurance and advice [62, 63, 66]. Our synthesis also suggests that education, exercise (strengthening, stretching, occupational exercise), manual therapy (manipulation) and soft tissue therapy (massage) are common components included in a multimodal care of care associated with superior outcomes for the management of lateral epicondylitis [62, 63, 65, 66]. On average, the intensity of multimodal care associated with superior outcomes included five visits offered over a five week period.

### Other systematic reviews

Systematic reviews that focus on the effectiveness of multimodal care are uncommon. We did not identify previous systematic reviews that specifically examined the effect of multimodal care for musculoskeletal disorders of the elbow, forearm, wrist and hand. However previous reviews combined the results of multimodal care with those of single interventions. Page et al. and Huisstede et al., reported that there is limited and very low quality evidence for combining exercise, splint and mobilisation interventions for the management of CTS [21, 74]. Further, Huisstede et al. concluded that there was no evidence for the effectiveness of chiropractic therapy which included manual therapy, massage, ultrasound, and a splint [21]. Two recent systematic reviews of lateral epicondylitis support our finding that exercise should be included as part of a multimodal program of care [75, 76]. However, these reviews had important limitations which included basing conclusions on studies with small sample sizes [21, 74–76] and not accounting for clustering in their analysis resulting in a unit of analysis error [21, 74].

### Strengths and limitations

Our systematic review has strengths. First, we developed a sensitive search strategy which was peer reviewed by a second librarian to minimize errors. Second, we used the Scottish Intercollegiate Guidelines Network (SIGN) criteria to ensure standardization of the critical appraisal process. We contacted authors to obtain further information in relation to study design. Finally, our conclusions are based on the best-evidence synthesis method to minimize the risk of bias associated with using low quality studies [44, 49].

Some limitations are noted in our review. First, we restricted our search to include articles in the English language, which may have excluded some relevant studies. However, other systematic reviews of clinical trials have also limited their search to the English language and this did not lead to biased results [77]. Other systematic reviews reported similar results when studying the effect of language-restrictions in conventional medicine [78–80]. Second, multimodal care is not a universally accepted search term in the rehabilitation literature. Although rehabilitation study interventions frequently include more than a single treatment modality, this information is often not clearly identified as a key word or presented in the study abstract. Thus, some studies, which employed multimodal interventions, may have been overlooked. Therefore, it is important that future trials of multimodal intervention correctly identify their interventions as multimodal. Third, the number of combinations of modalities in a multimodal program of care has no theoretical limit. This review reports on those combinations of modalities examined in the current literature. Future research of multimodal care should focus on multimodal programs of care which include modalities with demonstrated effectiveness. Fourth, we did not review qualitative studies exploring the lived experience of patients receiving multimodal care. We are therefore unable to comment on how patients valued and experienced their exposure to multimodal interventions. Although this is not a source of bias in our review, it is recommended that future systematic reviews consider examining qualitative studies to gain insight into the patients’ perspective of multimodal care. Finally, the reviewed studies were heterogeneous with respect to multimodal programs of care, outcomes measured and follow-up time points. This level of clinical heterogeneity did not allow pooling of results across studies through meta-analysis.

### Future research

Clinicians often combine modalities in a program of multimodal care, but little research is available to inform the best combination of modalities. Future research of multimodal programs of care should begin from the premise that only evidence-based modalities for the management of the musculoskeletal disorder of interest should be included in a defined program of care. Further, it is recommended that comparison groups which include the full program of multimodal care less one modality should be employed to ascertain the best combination of modalities. This systematic review identified low risk of bias studies examining persistent CTS and lateral epicondylitis. Research examining recent onset CTS and lateral epicondylitis are required to inform their management. Further, studies that address the effectiveness of multimodal care for the management of other musculoskeletal disorders of the elbow, forearm, wrist and hand are needed.

## Conclusions

Multimodal care reflects the combination of therapeutic interventions that are used by health care providers to manage patients with musculoskeletal disorders of the elbow, forearm, wrist and hand. Multimodal care for the management of persistent lateral epicondylitis may include education, exercise (strengthening, stretching, occupational exercise), manual therapy (manipulation) and soft tissue therapy (massage). The evidence did not support the use of multimodal care for the management of carpal tunnel syndrome. We did not identify low risk of bias studies for the management of other musculoskeletal disorders. Our systematic review highlights the need for further high quality studies to determine the effectiveness of multimodal care for musculoskeletal disorders of the elbow, forearm, wrist and hand.

## Additional file

Additional file 1:
**MEDLINE through OVID Search Strategy.** Description data: MEDLINE search strategies for musculoskeletal disorders of the elbow, forearm, wrist and hand. (DOCX 16 kb)

## Abbreviations

CDC: Centers for Disease Control and Prevention
CI: Confidence interval
CTSAQ: Carpal Tunnel Syndrome Assessment Questionnaire
CTS: Carpal tunnel syndrome
FSS: Functional Status Scale
*ĸ*: Kappa coefficient
MCID: Minimal clinically important difference
MeSH: Medical Subject Headings
NRS: Numeric Rating Scale
PRESS: Peer Review of Electronic Search Strategies
PRISMA: Preferred Reporting Items for Systematic Reviews and Meta-Analyses
PROSPERO: International Prospective Register of Systematic Reviews
PRTEE: Patient-rated Tennis Elbow Evaluation
RCT: Randomized controlled trial
SIGN: Scottish Intercollegiate Guidelines Network
SSS: Symptom Severity Scale
US: United States
USD: United States dollar
VAS: Visual analogue scale
