# Supplementary material for: Multimodal care for the management of musculoskeletal disorders of the elbow, forearm, wrist and hand: a systematic review by the Ontario Protocol for Traffic Injury Management (OPTIMa) Collaboration
Source: Chiropr Man Therap. 2016 Mar 7;24:8. doi: 10.1186/s12998-016-0089-8 (PMC4780149; doi:10.1186/s12998-016-0089-8)
Supplement: Additional file 1: — MEDLINE through OVID Search Strategy. Description data: MEDLINE search strategies for musculoskeletal disorders of the elbow, forearm, wrist and hand. (DOCX 16 kb) [file 12998_2016_89_MOESM1_ESM.docx]

**Additional file 1**

**MEDLINE through OVID Search Strategy**

1. exp "Sprains and Strains"/

2. Ulnar Nerve Compression Syndromes/

3. exp Median Neuropathy/

4. exp Arm Injuries/

5. exp Elbow/in [Injuries]

6. exp Hand Injuries/

7. Wrist Injuries/

8. Finger Injuries/

9. exp Tendinopathy/

10. Radial Neuropathy/

11. exp Ulnar Neuropathies/

12. Bursitis/

13. carpal tunnel syndrome.ab,ti.

14. radial tunnel syndrome.ab,ti.

15. (medial and (epicondylitis or epicondylosis or epicondylopathy)).ab,ti.

16. (lateral and (epicondylitis or epicondylosis or epicondylopathy)).ab,ti.

17. ((bicep* or bicipital or coracobrachialis) and (impingement or strain* or tear* or pain*)).ab,ti.

18. "bicep* tend?nitis".ab,ti.

19. (forearm* and (pain* or sprain* or strain* or injur* or impair*)).ab,ti.

20. (arm* and (pain* or sprain* or strain* or injur* or impair*)).ab,ti.

21. (wrist* and (pain* or sprain* or strain* or injur* or impair* or tendinitis)).ab,ti.

22. (hand* and (pain* or sprain* or strain* or injur* or impair*)).ab,ti.

23. (finger* and (pain* or sprain* or strain* or injur* or impair*)).ab,ti.

24. (elbow* and (pain* or sprain* or strain* or injur* or impair* or tendinitis)).ab,ti.

25. tennis elbow.ab,ti.

26. ((radial or ulnar or median) adj neuropath*).ab,ti.

27. bursitis.ab,ti.

28. "cubital tunnel syndrome*".ab,ti.

29. "overuse syndrome*".ab,ti.

30. (repetit* and (strain* or sprain* or injur* or disorder*)).ab,ti.

31. golfer* elbow.ab,ti.

32. (carpal and (pain* or sprain* or strain* or injur* or impair*)).ab,ti.

33. (thenar and (pain* or sprain* or strain* or injur* or impair*)).ab,ti.

34. (hypothenar and (pain* or sprain* or strain* or injur* or impair*)).ab,ti.

35. student* elbow.ab,ti.

36. pronator teres syndrome.ab,ti.

37. (De quervain* and (syndrome* or tenosynovit*)).ab,ti.

38. or/1-37

39. exp Hydrotherapy/

40. Laser Therapy, Low-Level/

41. Cryotherapy/

42. Magnetic Field Therapy/

43. exp Electric Stimulation Therapy/

44. exp Orthotic Devices/

45. exp Diathermy/

46. Hot Temperature/tu [Therapeutic Use]

47. Casts, Surgical/

48. Fluid Therapy/

49. Magnetics/tu [Therapeutic Use]

50. "Bedding and Linens"/

51. High-Energy Shock Waves/tu [Therapeutic Use]

52. Bed Rest/

53. Rest/

54. Self-Help Devices/

55. Restraint, Physical/

56. Gloves, Protective/

57. (cold and (therap* or pack* or compress or massage or immersion or soak or treatment or therap*)).ab,ti.

58. (ice and (therap* or pack* or compress or massage or immersion or soak or treatment or therap*)).ab,ti.

59. "glove*".ab,ti.

60. (heat* and (therap* or pack* or compress or massage or lamp or pad or bath or soak or tub or bottle or superficial or therapeutic)).ab,ti.

61. (hot and (therap* or pack* or compress or massage or lamp or pad or bath or soak or tub or bottle or superficial or therapeutic)).ab,ti.

62. ((shockwave* or shock wave* or shock-wave*) and (ultrasonic or therap* or radiation)).ab,ti.

63. "assistive device*".ab,ti.

64. (athletic and (tape or taping)).ab,ti.

65. (band* and (elbow or forearm or wrist)).ab,ti.

66. (braces or brace or bracing).ab,ti.

67. (cast or casts).ab,ti.

68. (collar or collars).ab,ti.

69. "cryotherap*".ab,ti.

70. diathermy.ab,ti.

71. (electric* and (stimulation or EMS or heating pad*)).ab,ti.

72. electroanalgesia.ab,ti.

73. (electrogalvanic stimulation or EGS).ab,ti.

74. (electromagnet* and (radiation or therap*)).ab,ti.

75. "electromodalit*".ab,ti.

76. electrotherapy.ab,ti.

77. "fluidotherap*".ab,ti.

78. galvanic stimulation.ab,ti.

79. (guard* and (teeth or night or mouth or wrist or knee)).ab,ti.

80. (high energy shock wave* or high-energy shock wave* or HESW).ab,ti.

81. (H-Wave Device Stimulation or HWDS).ab,ti.

82. "orthotic*".ab,ti.

83. "hydrotherap*".ab,ti.

84. infrared.ab,ti.

85. (interferential current* or ICS or IFC).ab,ti.

86. iontophoresis.ab,ti.

87. "kinesiotap*".ab,ti.

88. (laser* and (phototherapy or irradiation or biostimulation or light or therap*)).ab,ti.

89. "low level laser*".ab,ti.

90. (magnetic and (necklace* or therap* or bracelet*)).ab,ti.

91. Microcurrent Electrical Neuromuscular Stimulation.ab,ti.

92. "microwave*".ab,ti.

93. "moist air bath*".ab,ti.

94. muscle activation.ab,ti.

95. myofascial release.ab,ti.

96. (Neuromuscular Electrical Stimulation or NMES).ab,ti.

97. (paraffin and (treatment* or therap*)).ab,ti.

98. "passive modalit*".ab,ti.

99. "Percutaneous Electric* Nerve Stimulation".ab,ti.

100. "pillow*".ab,ti.

101. (pulsed and (electromagnetic or magnetic or radio frequency or energy)).ab,ti.

102. radiant light.ab,ti.

103. Russian stimulation.ab,ti.

104. "cushion*".ab,ti.

105. (short wave* or short-wave*).ab,ti.

106. (sling or slings).ab,ti.

107. (splint or splinting or splints).ab,ti.

108. "spray and stretch".ab,ti.

109. (tape or taping).ab,ti.

110. (transcutaneous electrical stimulation or TENS).ab,ti.

111. ultrasound.ab,ti.

112. vapocoolant spray.ab,ti.

113. "vibration therap*".ab,ti.

114. "warm compress*".ab,ti.

115. "wax treatment*".ab,ti.

116. parrafin wax.ab,ti.

117. whirlpool.ab,ti.

118. "water bath*".ab,ti.

119. or/39-118

120. Randomized Controlled Trials as Topic/

121. Controlled Clinical Trials as Topic/

122. exp case-control studies/

123. exp cohort studies/

124. double-blind method/

125. single-blind method/

126. Placebos/

127. randomized controlled trial.pt.

128. controlled clinical trial.pt.

129. meta-analysis.pt.

130. (meta analys* or meta-analys* or metaanalys*).ab,ti.

131. (cohort adj4 (study or studies or analys*)).ab,ti.

132. (cohort adj4 (study or studies or analys*)).ab,ti.

133. (random* adj4 (control* or clinical or allocat*)).ab,ti.

134. (case adj control*).ab,ti.

135. ((double or single) adj3 blind*).ab,ti.

136. "placebo*".ab,ti.

137. or/120-136

138. 38 and 119 and 137

139. limit 138 to (english language and humans and yr="1990 -Current")
